# Antitumor efficacy of a recombinant adenovirus encoding endostatin combined with an E1B55KD-deficient adenovirus in gastric cancer cells

**DOI:** 10.1186/1479-5876-11-257

**Published:** 2013-10-14

**Authors:** Li-xia Li, Yan-ling Zhang, Ling Zhou, Miao-la Ke, Jie-min Chen, Xiang Fu, Chun-ling Ye, Jiang-xue Wu, Ran-yi Liu, Wenlin Huang

**Affiliations:** 1 State Key Laboratory of Oncology in South China, Collaborative Innovation Center for Cancer Medicine, Sun Yat-sen University Cancer Center, Guangzhou 510060, China; 2 Department of Oncology, General Hospital of Guangzhou Military Command of PLA, Guangzhou 510010, China; 3 School of Biotechnology, Southern Medical University, Guangzhou 510515, China; 4 Department of Pharmacology, Pharmacy College of Ji-nan University, Guangzhou 510632, China; 5 Guangdong Provincial Key Laboratory of Tumor-targeted Drugs, Guangzhou Doublle Bioproducts Co., Ltd., Guangzhou 510663, China; 6 Guangzhou Enterprise Key Laboratory of Gene Medicine, Guangzhou Doublle Bioproducts Co., Ltd., Guangzhou, Guangdong 510663, China

**Keywords:** Endostatin, Adenovirus (Ad) vector, Oncolytic adenovirus (Ad), Viral-gene therapy, Gastric cancer

## Abstract

**Background:**

Gene therapy using a recombinant adenovirus (Ad) encoding secretory human endostatin (Ad-Endo) has been demonstrated to be a promising antiangiogenesis and antitumor strategy of in animal models and clinical trials. The E1B55KD-deficient Ad *dl*1520 was also found to replicate selectively in and destroy cancer cells. In this study, we aimed to investigate the antitumor effects of antiangiogenic agent Ad-Endo combined with the oncolytic Ad *dl*1520 on gastric cancer (GC) *in vitro* and *in vivo* and determine the mechanisms of these effects.

**Methods:**

The Ad DNA copy number was determined by real-time PCR, and gene expression was assessed by ELISA, Western blotting or immunohistochemistry. The anti-proliferation effect (cytotoxicity) of Ad was assessed using the colorimetry-based MTT cell viability assay. The antitumor effects were evaluated in BALB/c nude mice carrying SGC-7901 GC xenografts. The microvessel density and Ad replication in tumor tissue were evaluated by checking the expression of CD34 and hexon proteins, respectively.

**Results:**

*dl*1520 replicated selectively in GC cells harboring an abnormal p53 pathway, including p53 mutation and the loss of p14^ARF^ expression, but did not in normal epithelial cells. In cultured GC cells, *dl*1520 rescued Ad-Endo replication, and dramatically promoted endostatin expression by Ad-Endo in a dose- and time-dependent manner. In turn, the addition of Ad-Endo enhanced the inhibitory effect of *dl*1520 on the proliferation of GC cells. The transgenic expression of Ad5 E1A and E1B19K simulated the rescue effect of *dl*1520 supporting Ad-Endo replication in GC cells. In the nude mouse xenograft model, the combined treatment with *dl*1520 and Ad-Endo significantly inhibited tumor angiogenesis and the growth of GC xenografts through the increased endostatin expression and oncolytic effects.

**Conclusions:**

Ad-Endo combined with *dl*1520 has more antitumor efficacy against GC than Ad-Endo or *dl*1520 alone. These findings indicate that the combination of Ad-mediated antiangiogenic gene therapy and oncolytic Ad therapeutics could be one of promising comprehensive treatment strategies for GC.

## Introduction

Gastric cancer (GC) is one of the most common malignancies and a leading cause of cancer-related mortality worldwide, especially in Asian countries [1-3]. GC patients at early stage have no associated symptoms, and most of patients are initially diagnosed in an advanced stage, except those with GC at very early stage found predominantly by active screening programs in Asian countries [4]. Despite the recent development of new chemotherapy regimens and the introduction of biological therapies, the 5-year survival for advanced GC is still very low, and the median overall survival remains less than 1 year [5]. Therefore, the development of novel therapeutic approaches is crucial for improving the survival of GC patients.

The growth and metastasis of solid tumors are always accompanied by and depend on neovascularization [6-8]. Therefore, antiangiogenic therapy is an attractive strategy for the treatment of cancer [9-12]. Endostatin, a 20 kD C-terminal fragment of collagen XVIII composed of 184 amino acids, was previously considered the most potent angiogenesis inhibitor [13-16], and was rapidly moved to clinical trials [17]. However, the high instability and shorter serum half-life of the recombinant endostatin protein made it inappropriate or inconvenient for clinical application [18,19]. Daily administration is needed even for Endostar, a more stable product modified with a tag at the N-terminus [19,20]. Moreover, the long-term systemic delivery of a recombinant protein is an expensive, painful experience for patients and is cumbersome for medical staff. Antiangiogenic gene therapy can overcome these problems and represents a promising new approach for the treatment of cancer.

An adenoviral (Ad) vector encoding a secretory form of human endostatin (Ad-Endo, also referred to as E10A) has been demonstrated to inhibit tumor growth through antiangiogenic effects [21-24]. The results of preclinical trials showed that no notable toxicities were found in the experimental dogs after intramuscular injections of Ad-Endo at the doses equivalent to 30 and 7.5 times of the human curative dose once daily, 6 days/week, for 3 months [25]. In phase I clinical trials, the results showed that the treatment of solid tumor with Ad-Endo is likely a safe and promising approach [26,27]. The phase II clinical trial (ClinicalTrials.gov identifier, NCT00634595) has demonstrated that the addition of Ad-Endo improved the outcome of chemotherapy for the treatment of advanced nasopharyngeal carcinoma and head and neck cancer (Huang W, et al. unpublished data). However, Ad-Endo does not present a satisfactory therapeutic effect due to the limited expression of the endostatin protein, especially for tumors with large masses. Determining how to increase endostatin expression is a very important goal for further clinical trials.

Oncolytic Ad has been demonstrated to replicate selectively in cancer cells but not in normal cells [28-30]. We presumed that the selective replication of an oncolytic Ad could rescue the amplification of Ad-Endo genomic DNA and promote the expression of endostatin. In this study, we investigated the antitumor effects of the combined treatment of Ad-Endo and the oncolytic Ad *dl*1520 [31] on GC *in vitro* and *in vivo*. The results indicate that *dl*1520 enhanced the antiangiogenic effect of Ad-Endo by rescuing the replication of Ad-Endo, thereby dramatically increasing endostatin expression, when Ad-Endo in turn enhanced the oncolytic effect of *dl*1520 by reinforcing viral replication in GC cells.

## Materials and methods

### Cells, plasmids and transient transfection

The human GC cell line AGS (ATCC No. CRL-1739) [32], human embryonic kidney cell line 293 (ATCC No. CRL-1573) and human normal epithelial cell FHC (ATCC No. CRL-1831) [33] were obtained from the American Type Culture Collection (ATCC, Rockville, MD, USA). The human GC cell lines MGc80-3 [34] and SGC-7901 [35] were obtained from the Chinese Type Culture Collection. FHC cells were cultured in DMEM:F12 medium supplemented with 10% fetal bovine serum, extra 10 mM HEPES, 10 ng/ml cholera toxin, 0.005 mg/ml insulin, 0.005 mg/ml transferrin and 100 ng/ml hydrocortisone (Gibco, Paisley, UK). The other cells were all cultured in Dulbecco’s modified Eagle medium (DMEM) supplemented with 10% fetal bovine serum (Gibco, Paisley, UK) at 37°C with 5% CO_2_ and saturated humidity.

The plasmids expressing Ad2 E1A (pCD-E1A), E1B19k (pCD-E1B19k) or E1A+E1B19k (pCD-E1AB19k) were constructed by inserting the relevant gene fragments into the HindIII/EcoRI site of pcDNA3.1(+) vector (Invitrogen Corporation, Carlsbad, CA, USA). These gene fragments were amplified with the corresponding primers (Table 1) and *dl*1520 genomic DNA as the template. pCD-p14^ARF^ was constructed by subcloning full length of p14^ARF^ cDNA fragment (clone IMAGE: 6173590) into pcDNA3.1(+) vector. p14^ARF^ siRNA (sc-37622) was purchased from Santa Cruz Biotechnology, Inc (Santa Cruz, CA, USA). Plasmid or siRNA transfection was performed using the Effectene transfection reagent (Qiagen, Hilden, Germany) according to the manufacturer’s instructions.

**Table 1:** The sequence of primers used in this study

**Targets**	**Directions**	**Sequences**	**Notes**
**Real-time PCR Primers:**			
Ad-Endo (290 bp)	Forward	5′-TGACTGCCTCCAAGTAGGCTAGA-3′	Within the Endostatin fragment
	Reverse	5′-CCCAGATCCGCGTTAAGA-3′	At the junction of the poly_A signal and the Ad backbone
*dl*1520 (264 bp)	Forward	5′-TGTTTCCAGAACTGAGACGCAT-3′	E1B region (Ad2/2261-2281 nt)
	Reverse	5′ –TCTCATCGTACCTCAGCACCTT-3′	E1B region (Ad2 3330–3351 nt)
Total Ad (237 bp)	Forward	5′-TCGAAGCCGTTGATGTTGTG-3′	E2B (Ad2/7519-7538 nt or Ad5/7529-7548 nt)
	Reverse	5′- GGCCATAGGTCGCCAGTTTA-3′	E2B (Ad2/7519-7538 nt or Ad5/7529-7548 nt)
β-Actin (266 bp)	Forward	5′-CCTTTCCTTCCCAGGGCGTGAT-3′	At the intron 2-exon 3 junction
	Reverse	5′-CGGGCCACTCACCTGGGTCAT-3′	Within the exon 3
p53 (298 bp)	Forward	5′-GTGGTGGTGCCCTATGAG-3′	
	Reverse	5′-AGGAGCTGGTGTTGTTGG-3′	
p14^ARF^ (282 bp)	Forward	5′-CGCGAGTGAGGGTTTTCGT-3′	
	Reverse	5′-CAGCACCACCAGCGTGTCC-3′	
GAPDH (258 bp)	Forward	5′-AGAAGGCTGGGGCTCATTTG -3′	
	Reverse	5′-AGGGGCCATCCACAGTCTTC-3′	
**Cloning primers (for cloning into HindIII/EcoRI site of pcDNA3.1(+)):**			
Ad E1A	Forward	5′-ccc*aagctt*CGGGACTGAAAATGAGAC-3′	E1A gene (Ad2 548 nt – 1554 nt) (942 bp)
	Reverse	5′- ccg*gaattc*CAGGTTTACACCTTATGGC-3′	
Ad E1B19k	Forward	5′-ccc*aagctt*ATCTTGGTTACATCTGACCTC-3′	E1B19 kDa (Ad2 1690 nt – 2255 nt) (566 bp)
	Reverse	5′-ccg*gaattc*AGCCACCTGTACAACATTC-3′	
Ad E1A + E1B19k	Forward	5′-ccc*aagctt*CGGGACTGAAAATGAGAC-3′	E1A+E1B19 kDa (Ad2 548 nt – 2255 nt) (1708 bp)
	Reverse	5′-ccg*gaattc*AGCCACCTGTACAACATTC-3′	

### Recombinant adenoviruses (Ad) and infection

A replication-defective recombinant Ad vector encoding the secretory form of human endostatin (Ad-Endo) was generated in our lab as described previously [23,25]. The E1B55kD-deficient Ad (*dl*1520), also named Onyx-015 [31,36,37] was kind gift from Professor Arnold J. Berk (University of California-Los Angeles). The two viruses were both propagated in 293 cells, and the viral titers were determined using the hexon immunoassay with the BD Clontech™ Adeno-X Rapid Titer Kit (San Jose, CA, USA). For infection, gastric cells seeded 24 hours earlier were infected with Ad-Endo, *dl*1520 or Ad-Endo combined with *dl*1520 in serum-free DMEM for 2 hours, and then the infection medium was replaced with normal medium. The indicated time points post-infection correspond to the time after the medium change.

### Quantitative real-time PCR

For the measurement of the Ad DNA copy numbers, Ad-infected cells, including detached cells, were collected by scraping and centrifugation and then washed twice with PBS. The DNA was isolated using the Genomic DNA Mini Preparation Kit (Axygen, Hangzhou, China). The viral DNA copy numbers were measured by real-time PCR using the Platinum SYBR Green qPCR SuperMix-UDG (Invitrogen, Carlsbad, CA, USA). The primers for Ad-Endo, *dl*1520 or total Ad (β-actin was used as an internal control) are listed in Table 1. Real-time PCR was performed as follows: 50°C for 2 minutes, 95°C for 2 minutes and 40 cycles of 95°C for 15 seconds and 62°C for 1 minute. The viral DNA copy numbers are presented as relative values normalized to that of the internal control, 2^-ΔCt^. The change of DNA copy number is shown as the fold change relative to the DNA copy number at 0 hours post-infection.

For the quantitative detection of the mRNA levels of p53 and p14^ARF^, cells in the logarithmic growth phase were collected, and total RNA was isolated using Trizol Reagent (Invitrogen, Carlsbad, CA, USA). The RNA was then reverse transcribed into cDNA using GoScript™ Reverse Transcription System (Promega, Madison, WI, USA). Real-time PCR was performed as described above with special primer pairs (Table 1) (GAPDH was used as the internal control).

### Western blot analysis

Western blot analysis was performed as described previously [38]. Briefly, cell pellets were lysed with TNN-SDS buffer [38] at 4°C for 30 minutes followed by centrifugation (10,000 g for 10 minutes at 4°C) to remove the insoluble materials. The protein concentrations of the supernatants were measured using a Protein Assay kit. The proteins were then separated by sodium dodecyl sulfate-polyacrylamide gel electrophoresis, transferred to PVDF membranes, and probed with specific primary antibodies (p14^ARF^, Ad2 E1A and human actin antibodies from Santa Cruz Biotech., CA, USA; Ad2 E1B19K antibody from Calbiochem, Merck, Germany). After exposed to the primary antibodies, the membranes were reacted with relevant HRP-conjugated secondary antibodies, and the signals were detected with ECL reagents (Amersham Biosciences, Piscataway, NJ, USA) and x-ray film.

### Analysis of endostatin expression by ELISA

The culture supernatants of cells infected with Ad were collected at different time points and frozen at -80°C. The endostatin concentration was detected using a human endostatin ELISA kit (Shanghai ExCell Biology, Inc., Shanghai, China) according to the manufacturer’s instructions. The kit’s minimum detectable level is 30 pg/mL.

### *In vitro* Cytotoxicity assay

The cytotoxicity of Ad to GC cells was assessed by the MTT cell proliferation assay as previously described [38,39]. Briefly, cells were seeded in 96-well plates at a density of 3000 cells/well for 24 hours and then infected with Ad as described above, followed by incubation for 72 h. Viable cells were stained with MTT (Sigma-Aldrich, Shanghai, China) for 4 hours. The formazan crystals were dissolved with DMSO, and the optical density at 570 nm (OD_570nm_) was then measured using 630 nm as the reference wavelength.

### Animal models and *in vivo* antitumor activity

BALB/c-nu/nu mice (5-6 weeks old, 18-20 g) were obtained from the Experimental Animals Center, Sun Yat-sen University (Guangzhou, China). The mice were housed and fed under specific pathogen-free conditions according to protocols approved by the Sun Yat-sen University Institutional Animal Care and Use Committee. All experiments were performed in accordance with the Guidelines for the Welfare of Animals in Experimental Neoplasia. Pieces (approximately 1.5 mm in diameter) of SGC-7901 tumors, which were maintained by subcutaneous transplantation in nude mice, were subcutaneously transplanted into the flanks of mice to construct the xenograft model.

To assess the dynamic expression of endostatin *in vivo*, mice were injected intratumorally with Ad-Endo (5×10^8^ pfu/dose) or Ad-Endo plus *dl*1520 (5×10^8^ pfu/dose for each virus) when the xenografts reached an approximate diameter of 7 mm; 100 μL of PBS were used as the negative control. Blood plasma was sampled before and 1, 2, 3, 4, 6, 8, 13, and 21 days after virus administration (3 mice/group at each time point), and the endostatin concentration was determined by ELISA.

To assess the antitumor effects of Ad-Endo in combination with *dl*1520 *in vivo*, mice were randomly assigned to four groups (6–8 mice/group, half male and half female) when the xenografts reached 3–5 mm in diameter. The mice were treated by the intratumoral injection of 100 μL of PBS (control group), 5×10^8^ pfu of Ad-Endo (Ad-Endo group), 5×10^8^ pfu of dl1520 (*dl*1520 group), or 5×10^8^ pfu of Ad-Endo plus 5×10^8^ pfu of *dl*1520 (Ad-Endo+*dl*1520 group) (in 100 μL of PBS) per dose every 4 days for 4 consecutive weeks. Body weight and tumor size were measured every 4 days, and the tumor volumes were calculated according to the formula V = 0.52 × L × W^2^ (L, length; W, width) [14,23,39]. The tumor xenografts were weighed at the end point of the experiments.

### Immunohistochemical analysis

Tumor tissue was fixed in buffered formalin and embedded in paraffin. Sections (5 μM thick) were mounted on poly-L-lysine-treated slides, and immunohistochemical assays were performed to detect endostatin, CD34 and Ad hexon protein expression. Sections were probed with the following primary antibodies: mouse anti-Ad hexon McAb (MAB805, Chemicon/Millipore, Temecula, CA, USA), mouse anti-endostatin McAb (sc-32720, Santa Cruz, CA, USA), and rabbit anti-CD34 PcAb (BA3414, Boster, Wuhan, China). The protein expression was visualized with DAB using an EnVision™ detection kit (peroxidase/DAB, rabbit/mouse) (Gene Tech, Shanghai, China). The slides were counterstained with hematoxylin.

### Statistical analysis

All *in vitro* experiments were repeated at least three times, and the animal experiments were repeated at least two times. The data were analyzed with one-way ANOVA, two-way ANOVA or Student’s *t* test. P < 0.05 was considered statistically significant. The combined effect of the viruses was assessed with the Q value using Zheng-Jun Jin’s method [40]: Q=E_AB_/[E_A_+E_B_(1-E_A_)] (E_A_, E_B_ and E_AB_ indicate the effects of A, B and the combination of the two viruses). According to the Q value, the effect of the combination of two viruses can be classified as antagonistic (Q<0.85), additive (0.85<Q<1.15), or synergistic (Q>1.15).

## Results

### p53 pathway and oncolytic effects of *dl*1520 on GC cells

*dl*1520 has been reported to replicate in and lyse p53-mutant (mt-p53) cancer cells. To examine the oncolytic effects of *dl*1520 on GC cells and to determine if these effects depend on the p53 gene status, we first assessed the p53 gene status by sequencing the RT-PCR products of p53. The results showed that the MGc80-3 and SGC-7901 cell lines harbored a heterozygous mutant p53 (mt-p53) gene (codon 72 Pro → Arg), whereas the AGS and FHC cell line harbored a wild-type p53 (wt-p53) (data not shown).

**Figure 1:**
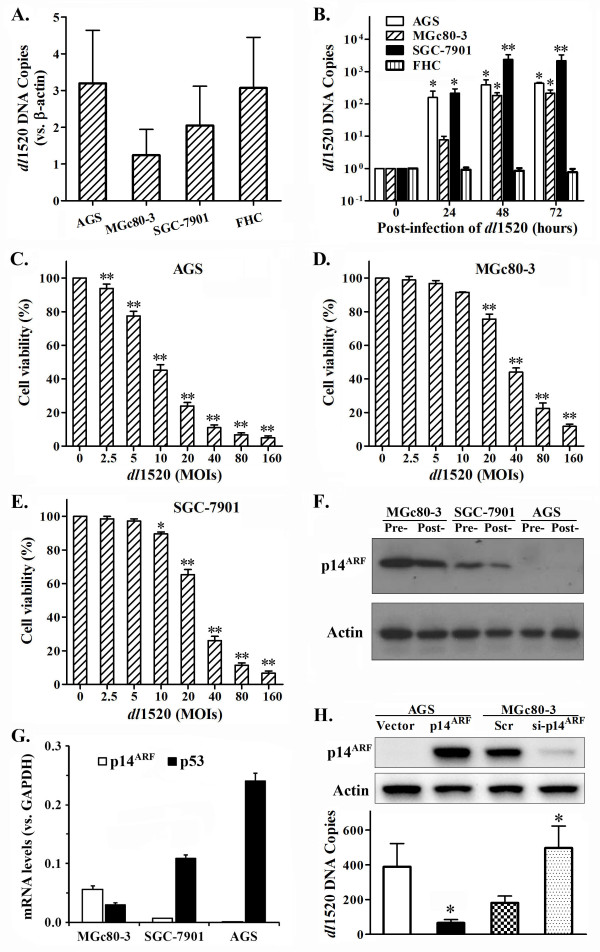
***dl*1520 inhibited the proliferation of GC cells by selectively replicating in and destroying the cancer cells. A, B)** The efficiencies of infection and replication of *dl*1520 in GC and normal cells. The infection efficiency **(A)** of *dl*1520 was shown as the *dl*1520 DNA copy number relative to β-actin at 0 hours after infection. The replication efficiency **(B)** of *dl*1520 was presented as the fold of the *dl*1520 DNA copy number at indicated time relative to that at 0 hours post-infection (One-way ANOVA, *p<0.05, **p<0.01 compared to that at 0 hours post-infection). **C~E)** The cytopathic effect (CPE) of *dl*1520 on GC cells. MTT cell proliferation assays were used to analyze the CPE of *dl*1520 on AGS **(C)**, MGc80-3 **(D)** and SGC-7901 **(E)** GC cells. The results are presented as the percentages of viable cells related to the negative control (one-way ANOVA, *p<0.05, **p<0.01 compared to that at 0 MOIs). **F)** Western blotting analysis of protein levels of p14^ARF^ in GC cells (Actin was used as the internal control). **G)** Quantitative RT-PCR analysis of the relative mRNA levels of p14^ARF^ and p53 (normalized to that of GAPDH). **H)** The replication of *dl*1520 after modifying the p14^ARF^ levels by knockdown or overexpression. AGS cells were transfected with pCD-p14^ARF^ plasmid (pcDNA3.1(+) as a negative control), and MGc80-3 cells were transfected with p14^ARF^ siRNA (si-p14^ARF^) (scrambled siRNA as a negative control). The cells were analyzed p14^ARF^ expression by Western blotting after 48 hours post-transfection (H upper). Or the cells were infected with 10 MOIs of *dl*1520 after 24 hours post-transfection, and *dl*1520 DNA copy numbers were analyzed in GC cells after 48 hours post-infection (normalized against that at 0 hours) (H lower). (Student’s *t* test, *p< 0.05 compared with their respective control).

To examine the replicative capacity of *dl*1520 in GC cells, the copy number of *dl*1520 DNA in GC cells was detected after *dl*1520 infection (FHC used as a normal cell control). The results showed that the copy numbers of *dl*1520 DNA at 0 hours after infection, indicating the infection efficiency of *dl*1520, are different, but not significantly, in the three GC cell lines and FHC cells (p> 0.05) (Figure 1A). The *dl*1520 DNA copy numbers increased greatly in AGS, MGc80-3 and SGC-7901 cells after infection compared to those at 0 hours post-infection, whereas *dl*1520 DNA copy number decreased slightly in FHC cells (Figure 1B). And the increased folds of *dl*1520 DNA in GC cells are not correlative to the infection efficiencies (data not shown). The titers of *dl*1520 after 48 hours post-infection were (4.11 ± 0.83) × 10^7^, (2.38 ± 0.41) × 10^7^, (1.27 ± 0.32) × 10^8^ and (9.17 ± 1.26) × 10^4^ pfu/mL in AGS, Mgc80-3, SGC-7901 and FHC cells respectively. These data suggested that *dl*1520 selectively replicated in GC cells but not in normal cells, and the replication of *dl*1520 was regardless of the p53 status in GC cells and infection efficiency.

To assess the cytopathic effect (CPE) due to *dl*1520 replication, an MTT cell proliferation assay was performed. The data indicated that *dl*1520 effectively lysed GC cells and inhibited their growth in a dose-dependent and p53-independent manner (Figure 1C, 1D and 1E). The findings are similar to those presented in Lee B et al’s report [41]. The replication of *dl*1520 and CPE resulted from *dl*1520 were stronger in AGS (wt-p53) and SGC-7901 (mt-p53) cells than those in MGc80-3 (mt-p53) cells, which is not consistent with the previous assumption.

For this reason, we assessed the expression of p14^ARF^, an important molecule in p53 pathway. The results showed that there was a high-level expression of p14^ARF^ at both the mRNA and protein levels in MGc80-3 cells but only a low level of expression in SGC-7901 cells; and p14^ARF^ expression was not detected in AGS cells (Figure 1F, 1G). These data indicated that the expression levels of p14^ARF^ were likely related to the selective replication of *dl*1520. To further verify the relation of p14^ARF^ with the replication of *dl*1520, we analyzed the replication of *dl*1520 after modifying the expression level of p14^ARF^ by knockdown or overexpression. The results showed that the knockdown of p14^ARF^ increased the *dl*1520 replication in MGc80-3 cells (p<0.05) whereas the overexpression of p14^ARF^ decreased the *dl*1520 replication in AGS cells (p<0.05) (Figure 1H). These findings suggested that the down-regulation or loss of p14^ARF^ expression played an important role in the oncolytic effects of *dl*1520 in GC cells. So, we concluded *dl*1520 replicated selectively in GC cells harboring an abnormal p53 pathway, including p53 mutation and the loss of p14^ARF^ expression.

### *dl*1520 rescues the replication of Ad-Endo in GC cells

**Figure 2:**
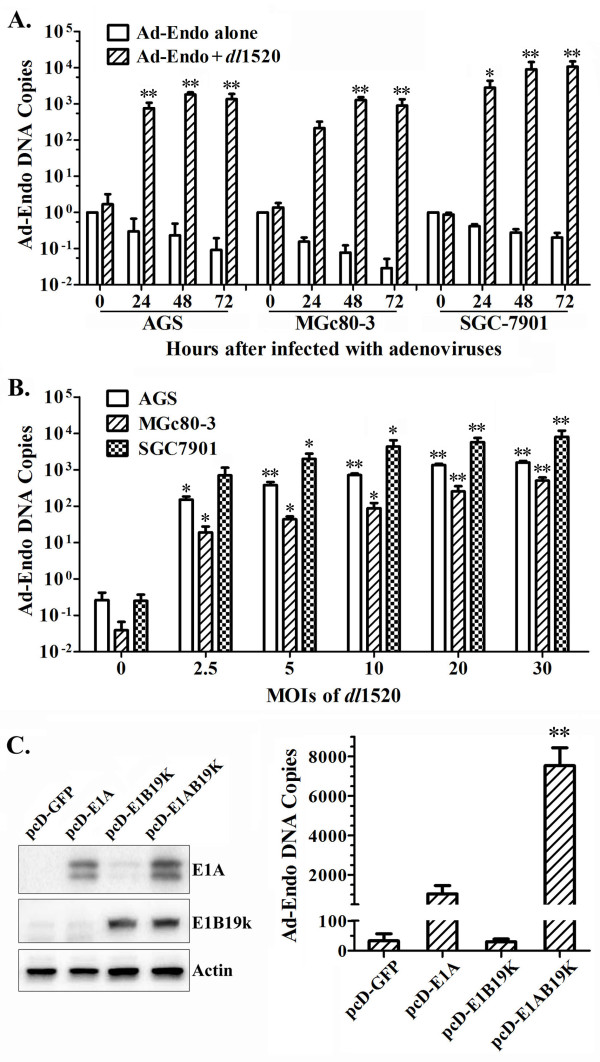
***dl*1520 rescued the replication of Ad-Endo in GC cells by supplying the E1A and E1B19k gene products.** The replication of Ad-Endo is presented as the increase in the Ad-Endo DNA copy number, which was determined by real-time PCR. The results are shown here as the fold change in the Ad-Endo DNA copy number at the indicated time points relative to that at 0 hours post-infection. **A)** Ad-Endo DNA copies in GC cells at different time points after infection with 10 MOIs of Ad-Endo alone or in combination with 10 MOIs of *dl*1520 (two-way ANOVA, *p<0.05, **p<0.01 compared to that at 0 hours post-infection). **B)** The Ad-Endo DNA copy number in GC cells at 48 hours after infection with 10 MOIs of Ad-Endo alone or in combination with increasing MOIs of *dl*1520 (one-way ANOVA, *p<0.05, **p<0.01 compared to that of *dl*1520 at 0 MOIs). **C)** The Ad-Endo DNA copy number in MGc80-3 cells transiently transfected with the E1A or/and E1B19k genes 48 hours after infection with 10 MOIs of Ad-Endo (one-way ANOVA, **p<0.01 compared to the control pCD-EGFP). The ectopic expression of the E1A and E1B19k genes in MGc80-3 cells was analyzed by Western blotting (actin was used as the internal control) (**C, Left**).

Ad-Endo is a replication-deficient recombinant Ad due to the deletion of the whole E1 region. We presumed that an oncolytic Ad could rescue the replication of Ad-Endo by providing some of the early proteins necessary for Ad replication. To test this hypothesis, the amount of Ad-Endo DNA in GC cells was a quantitatively determined by real-time PCR. The results showed that the Ad-Endo DNA copy number in GC cells infected with Ad-Endo plus *dl*1520 was greatly increased by more than 100-fold after infection, whereas the copy number decreased gradually in cells infected with Ad-Endo alone (Figure 2A). The rescue effects were enhanced with the higher *dl*1520 doses in a certain range (Figure 2B). These results indicated that the replication of Ad-Endo was likely rescued by *dl*1520 in GC cells.

### The rescue of Ad-Endo replication by *dl*1520 depends on the E1A and E1B19K proteins

To explore the mechanism involved in the rescue of *dl*1520 on Ad-Endo replication, the products of *dl*1520 E1 region genes (E1A, E1B19K) were ectopically expressed alone or together into MGc80-3 cells to investigate their effects on Ad-Endo replication. The results showed that the expression of the E1A (13S and 12S) and E1B19K together dramatically promoted Ad-Endo replication (p<0.01). The expression of E1A alone but not E1B19K also benefited Ad-Endo replication, but not significantly (Figure 2C). These data suggested that *dl*1520 rescued Ad-Endo replication by providing E1A and E1B19K proteins in GC cells.

### *dl*1520 promoted endostatin expression by Ad-Endo in GC cells

**Figure 3:**
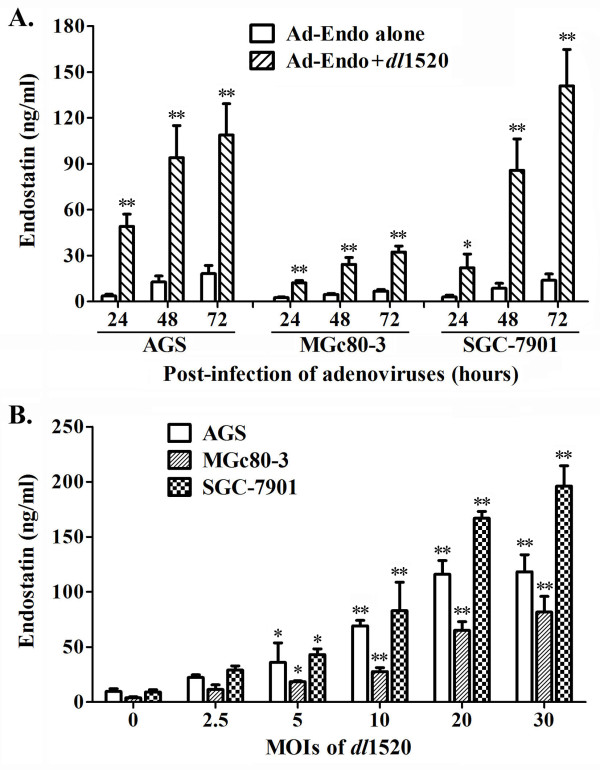
***dl*1520 promoted endostatin expression by Ad-Endo in GC cells.** GC cells were infected with Ad-Endo alone or combination with *dl*1520, and then the culture supernatants were collected at different time points. The endostatin concentrations were measured using a human endostatin ELISA kit (Shanghai ExCell Biology, Inc., Shanghai, China). The minimum detectable dose using this kit is 30 pg/mL. **A)** Endostatin concentrations in the culture supernatants of GC cells at different time points after infection with 10 MOIs of Ad-Endo alone or in combination with 10 MOIs of *dl*1520 (two-way ANOVA, *p<0.05, **p<0.01 compared to Ad-Endo alone). **B)** Endostatin concentrations in the culture supernatants at 48 hours after infection with 10 MOIs of Ad-Endo alone or in combination with increasing MOIs of *dl*1520 (one-way ANOVA, *p<0.05, **p<0.01 compared to that of *dl*1520 at 0 MOIs).

Since *dl*1520 can rescue the replication of Ad-Endo, we next asked whether *dl*1520 can enhance the antiangiogenic effects of Ad-Endo by promoting endostatin expression. Hence, we assessed the effect of *dl*1520 on endostatin expression by Ad-Endo in GC cells. The results showed that the endostatin concentrations in the supernatants from gastric cells infected with Ad-Endo plus *dl*1520 were much higher than those from cells infected with Ad-Endo alone (p<0.05 or p<0.01) (Figure 3A). In addition, the endostatin amount increased along with the increases in the *dl*1520 doses when cells were infected with Ad-Endo at a constant dose (10 MOIs) (Figure 3B). These results indicated that *dl*1520 promoted the expression of endostatin by rescuing Ad-Endo replication in GC cells.

### Ad-Endo enhanced the cytotoxic effects of *dl*1520 in GC cells

**Figure 4:**
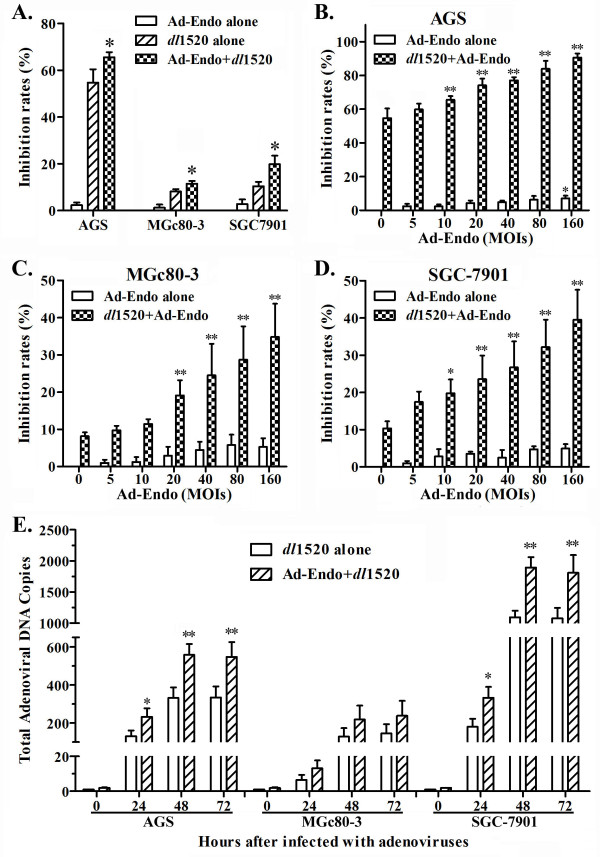
**Ad-Endo enhanced the cytotoxic effects of *dl*1520 in GC cells.** The *in vitro* cytotoxic effects were analyzed using the MTT cell proliferation assay. The proliferation activities of GC cells are represented as OD values at 570 nm (630 nm was used as the reference wavelength). The cytotoxic effects are represented as the inhibition rates (or inhibitory effects), and were calculated as follows: [(OD_negative control_ - OD_experiment_)/OD_negative control_]  ×  100%. **A)** The cytotoxic effects of 10 MOIs of Ad-Endo alone, 10 MOIs of dl1520 alone or the combination of the two on GC cells (one-way ANOVA, *p<0.05 compared with *dl*1520 alone and p<0.01 compared with Ad-Endo alone). **B~D)** The cytotoxic effects of 10 MOIs of dose *dl*1520 alone or in combination with increasing doses of Ad-Endo on AGS (**B**), MGc80-3 (**C**) and SGC-7901(**D**) GC cells (two-way ANOVA, *p<0.05, **p<0.01 compared to that of Ad-Endo at 0 MOIs). **E)** Ad replication in GC cells. Cells were infected 10 MOIs of dl1520 alone or in combination with 10 MOIs of Ad-Endo, and then the total Ad (Ad-Endo and *dl*1520) DNA copy number was detected by real-time PCR. Ad replication is presented as the increase in the total Ad DNA copy number. The results are shown here as the fold change in the total Ad DNA copy number at the indicated time points relative to that at 0 hours post-infection with *dl*1520 alone (two-way ANOVA, *p < 0.05, **p < 0.01 compared with *dl*1520 alone).

The above experiments demonstrated that *dl*1520 likely enhances the antiangiogenic effect of Ad-Endo by promoting endostatin expression. We then, in turn, assessed the effects of Ad-Endo on the cytotoxicity of oncolytic Ad. As shown in Figure 4A, 10 MOIs of Ad-Endo had almost no cytotoxicity, but this dose of Ad-Endo significantly enhanced the inhibitory effects of *dl*1520 on AGS, MGc80-3 and SGC-7901 GC cells (p<0.05). Moreover, the inhibitory effects of *dl*1520 increased along with increases in the Ad-Endo dose when the *dl*1520 dose was kept constant (10 MOIs), even though the same doses of Ad-Endo alone had little cytotoxicity (Figure 4B, 4C and 4D). Further investigations showed that the addition of Ad-Endo resulted in the increased replication of the total Ad, both *dl*1520 and Ad-Endo, especially in AGS and SGC-7901 cells (p<0.05 or p<0.01) (Figure 4E).

### *In vivo* antitumor effects of Ad-Endo combined with *dl*1520 on GC xenografts

**Figure 5:**
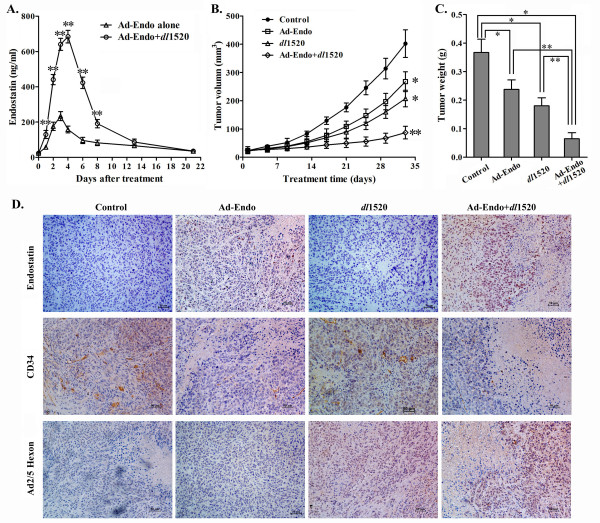
**Antitumor effects of Ad-Endo combined with *dl*1520 on GC SGC-7901 xenografts in nude mice. A)** The plasma concentration of endostatin in mice treated with a single intratumoral injection of Ad-Endo (5×10^8^ pfu) alone or in combination with *dl*1520 (5×10^8^ pfu). Blood plasma was sampled at the indicated time points, and the endostatin concentration was detected by ELISA (n=3) (two-way ANOVA, **p<0.01 compared to Ad-Endo alone). **B, C)** The growth inhibition of SGC-7901 xenografts by Ad-Endo or/and *dl*1520. **B)** The growth curves of SGC-7901 xenografts (two-way ANOVA, *p<0.01 compared to the control group; **p<0.01 compared to other three groups). **C)** Tumor weights (one-way ANOVA, *p< 0.01 compared with control group; **p<0.01 compared with the Ad-Endo+*dl*1520 group). **D)** Immunohistochemical assays of SGC-7901 xenografts to detect the expression of endostatin (upper), CD34 (middle) and the Ad hexon protein (lower) (×200). Brown staining indicates gene expression, and blue staining represents the cell nuclei. CD34, a marker of vascular endothelial cells, is used to assess the microvessel density in the tumor. Hexon staining indicates Ad replication.

To investigate the antitumor effects of combination treatment with Ad-Endo and *dl*1520, we first examined the endostatin concentration in the blood plasma to assess the endostatin expression by Ad-Endo alone or in combination with *dl*1520 in SGC-7901 GC xenografts in nude mice. As shown in Figure 5A, endostatin was expressed at the first day after intratumoral administration of Ad-Endo. And high levels of endostatin protein were detected in the blood 2 to 4 days and peaked at 3 days in mice treated with Ad-Endo alone. Notably, the plasma endostatin levels were much greater in nude mice treated with Ad-Endo plus *dl*1520 than those in mice treated with Ad-Endo alone (p<0.01). In addition, the duration of endostatin expression was prolonged in the combination group, and the plasma endostatin level peaked at 4 days after treatment (Figure 5A). These data suggested that *dl*1520 dramatically promoted endostatin expression by Ad-Endo in GC xenografts in nude mice, mirroring the results of the *in vitro* experiments. These results indicate that it is possible that *dl*1520 enhances the antitumor effect of Ad-Endo in animal models by promoting the expression of antiangiogenic factor endostatin.

As expected, the growth of SGC-7901 xenografts was significantly retarded by the administration of Ad-Endo or *dl*1520 alone (p<0.01) (Figure 5B, 5C). Moreover, the combined treatment with Ad-Endo and *dl*1520 showed much stronger antitumor effect on SGC-7901 xenografts in nude mice than either Ad-Endo or *dl*1520 alone (p<0.01) (Figure 5B, 5C). The inhibition rates of Ad-Endo alone, *dl*1520 alone or Ad-Endo plus *dl*1520 were 35.2%, 50.9%, and 82.4%, respectively (Figure 5C). According to Zheng-Jun Jin’s Q value (Q=1.21), Ad-Endo and *dl*1520 likely have synergistic antitumor effects on SGC-7901 xenografts in nude mice.

The immunohistochemical analysis for human endostatin also demonstrated that there was much stronger endostatin staining in tumor tissues treated with Ad-Endo plus *dl*1520 than in tissues treated with Ad-Endo alone, whereas no positive staining was observed in those tumor tissues treated with *dl*1520 alone or the medium control (Figure 5D upper row). Greater antiangiogenic effects were consequently found in the combination treatment group (Ad-Endo plus *dl*1520) than in Ad-Endo alone treatment group (Figure 5D middle row). These data suggested that *dl*1520 enhanced the antiangiogenic effects of Ad-Endo by promoting endostatin expression. Moreover, there were abundant Ad hexon proteins detected in the tumor tissues treated with *dl*1520 alone and those treated with Ad-Endo plus *dl*1520 (Figure 5D lower row), but not in paratumor normal tissue in nude mouse xenograft model (data not shown). These results indicated that *dl*1520 or *dl*1520 plus Ad-Endo selectively replicated in GC xenograft tissue but not in normal tissue, and the replication (oncolysis) played an important role in the antitumor effects of *dl*1520 combined with Ad-Endo on GC.

## Discussion

Gastric cancer (GC) is the second-most frequent cause of cancer-related death worldwide [3]. In most cases of GC, the p53 pathway is not functional due to the high frequency of p53 gene mutation or the loss of p14^ARF^ expression [42-45]. Therefore, oncolytic therapy with E1B55KD-deficient Ad is likely a potential treatment approach for GC. In this study, we found that *dl*1520, an E1B55KD- attenuated Ad, selectively replicated in and destroyed GC cells that have an abnormal p53 pathway, whereas it did not replicate in human normal epithelial cells (Figure 1) and paratumor normal tissue in nude mouse xenograft model (data not shown). Thus, *dl*1520 inhibited the growth of GC xenografts in nude mice (Figure 5). These results indicate that E1B55KD-deficient Ad, including *dl*1520 and Oncorine (H101) [46,47], an oncolytic Ad approved by the State Food and Drug Administration for the clinical application to treat squamous cell carcinoma of the head and neck, may be useful for the treatment of gastric cancer.

An important development in the field of tumor research was the establishment of the major role of angiogenesis in tumor development and the significance of antiangiogenic cancer therapies [48,49]. Among the 12 new anti-cancer drugs approved by the FDA in 2012, 4 are antiangiogenesis agents [50]. Endostatin is a novel potent inhibitor of angiogenesis with little toxicity, immunogenicity, and resistance [51]. Ad-Endo gene therapy can directly produce the endostatin proteins in a “factory” of mammalian cells. Benefiting from thorough post-translational modifications in mammalian cells, the endostatin proteins expressed by Ad-Endo have high bioactivity and stability [16,22-27,52]. Therefore, endostatin gene therapy is convenient in clinical application, and a cumbersome daily injection is no longer needed like the application of endostatin proteins. Up to date, Ad-Endo has completed its preclinical, phase I and phase II clinical trials [21-27,52,53] and started its phase III clinical trial on head and neck squamous cell carcinoma in China. However, Ad-Endo showed only a limited or moderate effect in previous clinical trials due to the limited increase in the endostatin concentration [26,27]. The antiangiogenic and antitumor effects are associated with the elevated local and circulating endostatin levels [54], therefore, it is important to increase the expression of transgenic endostatin.

The early proteins E1A and E1B are necessary for Ad replication [55]. Because of the deletion of the entire E1 region and part of the E3 region, Ad-Endo is a replication-defective recombinant Ad [23-25]. In this paper, we tried to promote Ad-Endo-directed endostatin expression by increasing the copy number of Ad-Endo with the help of tumor-selective replication of *dl*1520. We assumed that the selective replication of *dl*1520 may increase Ad-Endo-directed endostatin expression through rescuing the replication of Ad-Endo. So we firstly investigated whether *dl*1520 would promote the expression of endostatin in GC cells or not. The results showed that *dl*1520 rescued the selective replication of Ad-Endo in GC cells (Figure 2). The replication of Ad-Endo resulted in the increase of Ad-Endo DNA, which consequently caused a dramatic increase in the expression of endostatin by Ad-Endo in GC cells and a extension in the duration of endostatin expression in GC xenografts in nude mice (Figure 3 and Figure 5A). We further investigated the mechanism of *dl*1520 rescueing the replication of Ad-Endo, and found that the expression of the E1A (13S and 12S) and E1B19K genes together dramatically promoted Ad-Endo replication in MGc80-3 cells (Figure 2C). Therefore, we deduce that the expression of E1A and E1B19K proteins is likely a key event, by which *dl*1520 rescues the selective replication of Ad-Endo and strengthens the antiangiogenic and antitumor effect of Ad-Endo on GC cells.

To the best of our understanding, oncolytic Ad exerts the antitumor effect through viral replication and the consequent lysis of tumor cells. Since the replication of Ad-Endo was observed in GC cells co-infected with *dl*1520, theoretically, Ad-Endo should in turn contribute to the oncolytic effect. Intriguingly, in our study, we also found that the combination of Ad-Endo and *dl*1520 resulted in the reinforcement of the oncolytic effect, which may be attributed to the increase in Ad replication reflected by the increased total Ad DNA copy number (Figure 4). That is, the antiangiogenic agent Ad-Endo and the oncolytic virus *dl*1520 promote their anti-GC effects each other. This hypothesis was confirmed by the combined treatment experiments against SGC-7901 xenografts in nude mice in this paper. There was a synergistic antitumor effect between *dl*1520 and Ad-Endo for the treatment of the SGC-7901 xenografts in nude mice. This effect resulted from increased antiangiogenic effects and enhanced oncolysis (Figure 5). Considering that E1B55kD-delected adenovirus H101 has been approve to treat solid tumors in China, and Ad-Endo has been in its phase III clinical trial and will likely be applied to cancer treatment soon, our findings provided an experimental basis for combined application in future.

In Ad vector-based gene therapy, it is always considered that strong anti-Ad vector immune response induced by multiple injections of recombinant Ad may severely hinder the therapeutic efficiency. Local (intratumoral) administration may be one of approaches to minish this negative effect. We have previously demonstrated that multiple intratumoral injections of Ad-Endo resulted in strong anti-Ad immune response in immuno-competent mouse model, but did not lead to continuous increases of Ad neutralizing antibodies [23]. Thus, the host immune response to the vector decreased serum endostatin levels slightly upon readministration, but the endostatin concentrations were above the efficient treatment concentration, anti-angiogenic effect could still be achieved during 5 courses of endostatin gene therapy [23]. Another report showed that intratumoral administration of recombinant Ad also inhibited the tumor growth by activation of direct and indirect immune response to exert “bystander effects” in immuno-competent individual [56]. In this study, we used athymic nude mouse as animal models, which lack of T cell response (including Th-helped B cell response). Only nonspecific immune response, such as natural killer cells, will be activated after multiple intratumoral injections of Ad. So, the negative effects of the immune response induced by multiple intratumoral injections of Ad vectors should be weaker than in immuno-competent mouse model. Considering that immune inhibition is often observed in cancer patients, especially in advanced cases, the negative effects of the immune response will be slight in future clinical application, and can be ignored compared to the benefits.

Local (or intratumoral) administration of Ad-based gene therapy was often limited to apply in malignancies on body surface, such as melanoma, hear and neck tumors, although local administration will minish a part of the impairment effects of immune response. However, the utilization of modern imaging technology extends the application of intratumoral approach to lung, gastric, colorectal, liver cancers etc. Local application of therapeutic Ad vectors also likely benefits GC patients via administering under gastroscope guidance, or applied directly into ascites in advanced GC patients.

## Conclusions

Our results show that the E1B55KD-attenuated Ad *dl*1520 can selectively replicate in and destroy GC cells and that it promotes the antiangiogenic effects of Ad-Endo through rescuing the replication of Ad-Endo and consequently increasing the expression of endostatin in GC cells. Furthermore, Ad-Endo enhances the oncolytic effect of the E1B55KD-attenuated Ad *dl*1520 by reinforcing viral replication. The antiangiogenic agent Ad-Endo and the oncolytic Ad *dl*1520 have synergistic antitumor effects on gastric cancer. These findings indicate that the use of Ad-based oncolytic virus therapeutics in combination with Ad-based antiangiogenic gene therapy is likely one of promising approaches for the comprehensive treatment of gastric carcinoma.

## Abbreviations

Ad: Adenovirus or adenoviral; Ad2: Adenovirus type 2; Ad-Endo: A replication-defective adenovirus encoding a secretory form of human endostatin; cDNA: Complementary DNA; CPE: Cytopathic effect; DMEM: Dulbecco’s modified eagle medium; ELISA: Enzyme-linked immunosorbent assay; GC: Gastric cancer; MOI: Multiplicity of infection; mt-p53: Mutant p53 gene; MTT: (3-(4,5-dimethylthiazol-2-yl)-2,5-diphenyltetrazolium bromide; PCR: Polymerase chain reaction; wt-p53: Wild- type p53 gene.

## Competing interests

The authors declare that they have no competing interests.

## Authors’ contributions

LL performed the viral DNA copy assay, determined the endostatin concentrations, conducted the antitumor animal experiments, collected and analyzed all of the results, and outlined and drafted the article. YZ generated the Ad stocks, performed the Western blotting analysis, assessed the endostatin expression levels in the experimental animals, and collected and analyzed the results. LZ constructed the plasmids and performed the knockdown and overexpression experiments. XF and MK carried out immunohistochemical assays. JC and JW performed the p53 cDNA sequencing and assessed the expression levels of p53 and p14. CY performed and validated the statistical analysis. RL and WH conceived and coordinated the work and helped draft the manuscript. All authors have read and approved the final manuscript.
